# Inducible nitric oxide synthase is involved in the modulation of depressive behaviors induced by unpredictable chronic mild stress

**DOI:** 10.1186/1742-2094-9-75

**Published:** 2012-07-06

**Authors:** Yun-Li Peng, Yu-Ning Liu, Lei Liu, Xia Wang, Chun-Lei Jiang, Yun-Xia Wang

**Affiliations:** 1 Department of Nautical Medicine, Lab of Stress Medicine, Second Military Medical University, 800 Xiangyin Road, Shanghai, 200433, P R China

**Keywords:** Depressive behavior, inducible nitric oxide synthase, unpredictable chronic mild stress

## Abstract

**Background:**

Experiences and inflammatory mediators are fundamental in the provocation of major depressive disorders (MDDs). We investigated the roles and mechanisms of inducible nitric oxide synthase (iNOS) in stress-induced depression.

**Methods:**

We used a depressive-like state mouse model induced by unpredictable chronic mild stress (UCMS). Depressive-like behaviors were evaluated after 4 weeks of UCMS, in the presence and absence of the iNOS inhibitor *N*-(3-(aminomethyl)benzyl)acetamidine (1400 W) compared with the control group. Immunohistochemistry was used to check the loss of Nissl bodies in cerebral cortex neurons. The levels of iNOS mRNA expression in the cortex and nitrites in the plasma were measured with real-time reverse transcription PCR (RT-PCR) and Griess reagent respectively.

**Results:**

Results showed that the 4-week UCMS significantly induced depressive-like behaviors, including decreased sucrose preference in a sucrose preference test, increased duration of immobility in a forced swim test, and decreased hole-searching time in a locomotor activity test. Meanwhile, in the locomotor activity test, UCMS had no effect on normal locomotor activities, such as resting time, active time and total travel distance. Furthermore, the levels of iNOS mRNA expression in the cortex and nitrites in the plasma of UCMS-exposed mice were significantly increased compared with that of the control group. Neurons of cerebral cortex in UCMS-exposed mice were shrunken with dark staining, together with loss of Nissl bodies. The above-mentioned stress-related depressive-like behaviors, increase of iNOS mRNA expression in the cortex and nitrites in the plasma, and neuron damage, could be abrogated remarkably by pretreating the mice with an iNOS inhibitor (1400 W). Moreover, neurons with abundant Nissl bodies were significantly increased in the 1400 W + UCMS group.

**Conclusions:**

These results support the notion that stress-related NO (derived from iNOS) may contribute to depressive-like behaviors in a mouse model, potentially concurrent with neurodegenerative effects within the cerebral cortex.

## Background

The challenges of modern society are largely responsible for generating stress in human beings. Stress in turn engenders multiple neurochemical, neurotransmitter and hormonal changes. Studies carried out with some stress protocols (physical, psychological or mixed) show a proinflammatory response in the brain and other systems, mainly characterized by a complex release of several inflammatory mediators [1]. In recent years, inflammation has been implicated in chronic psychiatric disorders. Cytokines such as interleukin (IL)-1β and IL-6 are elevated in the serum of depressed patients [2]. Therefore, stress has been one of the most important pathogenic factors in several neuropsychiatric diseases such as depressive disorder. And stress exposure modifies the onset and evolution of some neurological diseases [1]. According to a report from the World Health Organization (WHO), depression is the fourth highest contributor to the global burden of disease and is predicted to be in second place by 2020 [3]. Therefore, it is important to reveal the mechanism of depression. However, it is still unknown that how chronic stress induces or precipitates depression.

Nitric oxide (NO), a free gaseous signaling molecule, is involved in the regulation of the nervous and immune system. It has been suggested NO is involved in depression and stress [4]. There are three genetically different isoforms of nitric oxide synthase (NOS) that account for NO production: neuronal nitric oxide synthase (nNOS) being the isoform first identified in neurons, endothelial nitric oxide synthase (eNOS) being the isoform first identified in endothelial cells, and inducible nitric oxide synthase (iNOS), which can be synthesized following induction by proinflammatory cytokines or endotoxins [5]. The NOS enzymes are widely distributed within the mammalian brain. NOS-positive neurons are located in the hippocampus, cerebral cortex and other encephalic regions [6]. Various studies suggest the involvement of nNOS in the pathophysiological mechanism of depression-like behavior in rodents [7-10]. Over the last two to three decades, the ‘inflammatory depression hypothesis’ has attracted great attention. Chronic inflammation is often associated with clinical depression [11-15]. Chronic stress is associated with dysregulated immunity by overactivating the immune system, leading to low-grade inflammation [16]. iNOS is inflammation inducible and plays no role in the brain under normal physiological conditions. However, under pathological conditions, iNOS may become important. Therefore, we chose iNOS as our focus to identify whether and how NOS is involved in the pathogenesis of the depressive behavior induced by chronic stress exposure.

Converging lines of research suggest that both functional alterations and structural changes in the volume of the hippocampal complex play roles in the pathophysiology of depressive disorder [17]. And the effects of stress on the hippocampus are clear. The hippocampus is often considered to be the classical region to assess morphological effects related to depression. However, morphological plasticity has been less intensively studied in the cortex than in the hippocampus under stressed conditions. Furthermore, clinical and experimental evidence suggest the cortex is pathophysiologically related to depression. However, it is unknown what roles the cortex plays in stress-initiated depression. We selected the cortex for morphological assessment in our present study to investigate its potential importance in stress-related depression.

Considering that chronic stress can induce inflammatory responses and depressive behaviors in animals, and iNOS is inflammation inducible, we hypothesize that iNOS plays a critical role in stress-related depression. If the above assumption is true, the levels of iNOS and NO should be changed in stress-exposed animals. Additionally, an inhibitor of iNOS should block the alteration of iNOS and NO, followed by the recovery of depressive behaviors induced by stress exposure. The potential mechanism underlying the antidepressant effects of iNOS inhibitor may be neuron protection. Therefore, the present study was performed to verify the above hypothesis in a step-by-step manner.

## Methods

### Reagents

Quantscript cDNA RT kit (catalogue no. KR-103), reverse transcriptase kit and TRIzol reagent were purchased from Tiangen Biotech (Beijing, China). Real-time reverse transcription PCR (RT-PCR) primers for iNOS (catalogue no. M301149, M301150) and glyceraldehyde-3-phosphate dehydrogenase (GAPDH; catalogue no. M301155, M301156) were all obtained from Sangon Biotech (Shanghai, China). Total nitric oxide assay kit and iNOS inhibitor *N*-(3-(aminomethyl)benzyl)acetamidine (1400 W) were obtained from Beyotime Institute of Biotechnology (Jiangsu, China). Other reagents and chemicals were obtained from Sigma Aldrich (St. Louis, MO, USA).

### Animals and chronic unpredictable mild stress model

Male, 9-week-old BALB/c mice weighing 20 to 25 g (Animal Centre, Second Military Medical University, China) were used in this study. All procedures were approved by the local animal care committees in accordance with related regulations and laws. Before the onset of experiments, 1 week was allowed for the mice to adapt to their new circumstances, and another 2 weeks to adapt to the sucrose solution. Next, the mice were split into three groups: a non-stressed group, a UCMS exposed (stressed) group, and a UCMS exposed + 1400 W group. Non-stressed mice were group housed under standard laboratory conditions (12-h light: 12-h dark cycle, lights on at 07:00 AM; temperature: 22 ± 1°C; humidity: 52 ± 2%) in a separate room. Stressed mice were isolated in individual cages. All animals received food and water *ad libitum*, except for during the sucrose preference test. The UCMS regimen was based on the procedures described by other researchers, with minor modifications [18]. Briefly, UCMS-exposed mice were subjected to various stressors in a chronic, inevitable and unpredictable way according to a random schedule for 4 weeks. The stressors were: damp bedding for 12 h; 45° cage tilting for 14 to 18 h; continuous light on (24 h); water and food deprivation for 14 to 18 h; strong level shaking for 10 minutes; ice-cold swimming for 5 minutes; 45°C oven for 5 minutes; confinement in a tube for 2 h. Random stressors (as shown in Table 1) were applied on purpose, at random times of both night and day, in order to be completely unpredictable. All mice in the stress group were exposed to the same single stressor simultaneously in 1 day. No single stressor was applied for 2 days consecutively. During the whole process of stress, each stressor was applied two or three times. Body weight and sucrose preference were assessed weekly. Drug or vehicle treatments started on day 7 and were stopped the day after the end of UCMS (day 28). 1400 W was injected intraperitoneally at 10 mg/kg three times a week as according to a previous report [19]. The control group received saline solution of the same volume as that of 1400 W.

**Table 1:** Unpredictable chronic mild stress procedure

Week	Monday	Tuesday	Wednesday	Thursday	Friday	Saturday	Sunday
First week	8:00 AM to 10:00 PM: tilted cage	10:00 AM to 12:00 AM: cage shaking for 10 minutes	17:00 PM to next day: damp bedding	9:00 AM to 11:00 AM: swimming at 4°C for 5 minutes	15:30 PM to 17:30 PM: confinement in tube for 2 h	22:00 PM to next day: food and water deprivation	21:00 PM to 22:00 PM: sucrose preference test
Second week	17:30 PM to next day: persistent illumination	9:00 AM to 11:00 AM: 45°C oven for 5 minutes	18:00 PM to next day: tilted cage	13:30 PM to 15:30 PM: confinement in tube for 2 h	19:00 PM to next day: damp bedding	20:00 PM to next day: food and water deprivation	19:30 PM to 20:30 PM: sucrose preference test
Third week	10:30 AM to 12:30 PM: confinement in tube for 2 h	17:00 PM to next day: damp bedding	8:00 AM to 10:00 PM: tilted cage	19:30 PM to next day: persistent illumination	10:00 AM to 12:00 AM: cage shaking for 10 minutes	21:00 PM to next day: food and water deprivation	20:00 PM to 21:00 PM: sucrose preference test
Fourth week	19:00 PM to 21:00 PM: 45° C oven for 5 minutes	8:00 AM to 10:00 PM: tilted cage	13:30 PM to 15:30 PM: confinement in tube for 2 h	9:00 AM to 11:00 AM: cage shaking for 10 minutes	17:30 PM to next day: persistent illumination	23:00 PM to next day: food and water deprivation	22:30 PM to 23:30 PM: sucrose preference test

### Behavioral tests

#### Sucrose preference test

Anhedonia was measured by preference for a sucrose solution over water, using a two-bottle free choice method [20]: each animal was presented simultaneously with two bottles, one containing 1% sucrose solution (w/v), the other containing tap water. Blunted sucrose intake in this test is proposed to reflect impaired sensitivity to reward and model anhedonia, a core symptom of major depression [21]. Tap water and 1% sucrose solution were placed in premeasured bottles in the cages, and fluid intake was monitored for 1 h. Both bottles were removed and weighed after 1 h. Mice were given access to sucrose solution for 2 weeks preceding the experimental procedures to adapt to this taste. Sucrose preference tests were timetabled during the dark phase (19:00 PM to 20:00 PM) in the home cage. Mice were denied food and water for about 20 h before each sucrose preference test. The baseline preference test was performed before the onset of stress, and preference tests were then conducted weekly throughout the UCMS period. Sucrose preference was evaluated via the sucrose uptake rate, namely, the ratio of volume of sucrose consumption to the volume of sucrose consumption plus tap water consumption (sucrose preference = sucrose consumption/(sucrose consumption + water consumption) × 100%).

#### Locomotor activity test

On the subsequent day after the last sucrose preference test, a locomotor activity test was carried out during the night. The locomotor activity test is used to measure spontaneous activity in rodents. The apparatus consisted of a dark rectangular box with a square floor, divided into small rectangular units and holes. A single mouse was gently placed in the center of the box for 30 s of adaptation, and then allowed to freely explore the area for 5 minutes. All behaviors including the number of activities, the resting and active time, hole-searching time, and so on, were recorded automatically by DigBehv animal behavior analysis software (Jiliang Software Technology, Shanghai, China) during the 5 minutes. After each test, the floor was cleaned thoroughly with 75% alcohol solution to eliminate possible bias due to odors left by previous mice [22].

#### Forced swim test

The forced swim test (FST) was performed following the locomotor activity test at night-time, conducted as described previously [23]. Briefly, each mouse was placed individually in a transparent cylindrical polypropylene tank (40 cm height × 30 cm diameter) containing 35 cm of water at 25 ± 1°C, without the possibility of escaping. Mice were forced to swim in the water for 6 minutes. A mouse was judged immobile when it floated in an upright position, and could only move slowly to keep its head above water. The duration of immobility during the final 5 minutes of the test was recorded. This immobile posture reflects a state of behavioral despair or helplessness [24]. Mice were dried immediately and returned to their home cages after the swimming test.

### Sample collection

After the behavioral tests, three mice from each group were deeply anesthetized and perfused with 4% paraformaldehyde for subsequent Nissl staining. The other animals were anesthetized and killed; blood was collected and brains were removed. Blood, anticoagulated with 1.5% EDTA was centrifuged at 12,000 rpm for 10 minutes, and then the supernatant was collected. All these samples were stored at −80°C for further analysis.

### RNA extraction and reverse transcription

Total RNA was extracted from the brain tissue using TRIzol reagent. Total mRNA (1 μg) was reverse transcribed using Quantscript cDNA RT Kits according to the manufacturer’s manual. Briefly, RNA (1 μg) was pretreated with DNA-free DNase treatment and removal reagents. RNA samples were incubated with a mixture consisting of containing dNTPs, random primers, 10× RT mix, Quant Reverse Transcriptase, a reverse transcriptase and RNase-free water to a final volume of 10 μl at 37°C for 1 h.

### Real-time RT-PCR

cDNA was used for quantification of mRNA by real-time RT-PCR. Real-time RT-PCR was performed on an Applied Rotor-Gene 3000 (Corbett Research, China)under the following conditions: iNOS and GAPDH for 40 cycles at 94°C for 30 s, 63°C for 60 s, and 72°C for 90 s. Relative quantitative measurements of target gene levels were performed using the ΔΔCt method, where Ct is the threshold concentration. GAPDH was used as endogenous control to normalize gene expression data, and an RQ value was calculated for each sample. RQ values are presented as fold change in gene expression relative to the control group, which was normalized to 1. The following oligonucleotides were used as primers: iNOS (forward, 5'-GACTGCACAGAATGTTCCAG-3'; reverse, 5'-TGGCCAGATGTTCCTCTATT-3'), GAPDH (forward, 5'-TCCCTCAAGATTGTCAGCAA-3'; reverse, 5'-AGATCCACAACGGATACATT-3') [25].

### Total NO production assay

Total NO production was estimated by measurement of the accumulation of nitrite and nitrate in plasma spectrophotometrically using the Griess reagent by Total Nitric Oxide Assay Kit (Beyotime, Jiangsu, China). Nitrate was measured after enzymatical conversion to nitrite by nitrate reductase. Nitrite is the stable reactive end production of NO. Briefly, 60 μl of each sample supernatant was mixed with an equal volume of dilution buffer in duplicate wells of a 96-well plate at room temperature. The mixture was incubated with 5 μl of nicotinamide adenine dinucleotide phosphate (NADPH), 10 μl of flavin adenine dinucleotide (FAD) and 5 μl of nitrate reductase for 15 minutes at 37°C. Then, 10 μl of lactate dehydrogenase (LDH) buffer and 10 μl of LDH were added in the above reaction buffer for another 5 minutes at 37°C. Finally, 50 μl of Griess reagent I and 50 μl of Griess reagent II were mixed into all the above wells before incubation for 10 minutes. Optical density at 540 nm was measured with an OPTImax multiplate reader. Concentrations were calculated by comparing absorptions with those of a standard curve (50, 20, 10, 5 and 2 μM sodium nitrite).

### Immunohistochemistry

#### Nissl staining

After behavioral tests, three mice from each group were deeply anesthetized and perfused through the left heart ventricle with 4% paraformaldehyde in 0.1 M phosphate buffer (pH 7.4). The brain tissues were removed and post fixed immediately in the same fixative solution at 4°C for 48 h. Next, the tissues were dehydrated and immersed in paraffin, cut into 4 μm sections, mounted on slides. For Nissl staining, the sections were hydrated in 1% toluidine blue at 50°C for 20 minutes. After rinsing with double distilled water, they were dehydrated and mounted with permount. The cortex was captured and cell numbers were quantitatively analyzed with Imaging Pro-Plus (LEIKA DMLB).

#### Cell counting

To estimate the number of undamaged neurons in the cortex after treatments, photos of immunohistochemical slides were taken using a digital camera connected to a microscope at 100× and 400× magnification. Neurons with round cell bodies, visible nucleus and abundant Nissl bodies were considered undamaged, while Nissl-positive cells with dark staining in which the nucleus were not discernable were considered damaged [26]. Detailed performance and measurements were as previously described [27].

### Statistical analysis

For multiexperimental group analysis, data were analyzed using a one-way analysis of variance (ANOVA), followed by a *post hoc* pairwise multiple comparison using Fisher’s least significant difference test if the interaction was significant. The time course of sucrose preference was analyzed by repeated measures ANOVA. Statistical significance was determined as *P* < 0.05. All data are presented as the mean ± SEM.

## Results

To explore whether and how depressive behavior was induced by UCMS, mice were treated with UCMS for 4 weeks in the presence and absence of the iNOS inhibitor 1400 W. Depressive behaviors were measured and analyzed, and iNOS expression and neuron viability were also assayed.

### Decrease of sucrose preference was induced by UCMS in a time-dependent manner

**Figure 1:**
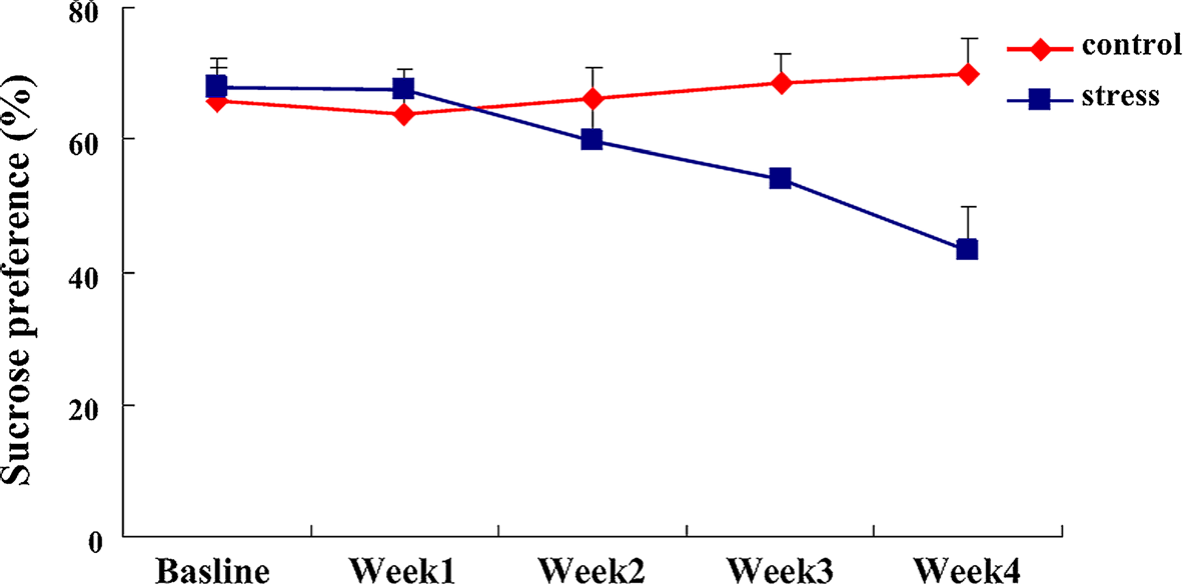
**Unpredictable chronic mild stress (UCMS) induced the decrease of sucrose preference in a time-dependent manner.** UCMS-exposed mice were subjected to UCMS for 4 weeks. Sucrose preference was evaluated via sucrose uptake rate, namely, ratio of volume of sucrose consumption to volume of sucrose plus tap water consumption. Data are mean ± SEM (n = 7 for control; n = 6 for stress).

The sucrose preference test is used to validate animal models of depression, especially chronic mild stress (CMS). When given a choice between sweetened solutions and tap water, rodents prefer to consume sweetened solutions [28,29]. Anhedonia, often reflecting depression, can be demonstrated by the reduction in sucrose intake and sucrose preference, compared to the baseline, or that of the control group. So the change of sucrose preference in UCMS-treated mice was measured and analyzed. A time course of sucrose preference was performed. Mice were subjected to various stressors according to a ‘random’ scheme for 4 weeks. The basal sucrose preference was performed at the beginning of stress exposure. Next, real-time sucrose preference was measured at each weekend to estimate the effect of stress. Figure 1 showed that the sucrose preference of UCMS-exposed mice decreased in a time dependent manner, and reached the minimum at the third to fourth week, as shown by a significant treatment effect (F_(1,11)_ = 8.60, *P* < 0.05). Therefore, we decided to use a 4-week UCMS treatment in subsequent experiments. The sucrose preference of stress-exposed mice began to decrease at the end of the second week. So the inhibitor of iNOS was administrated at the beginning of the second week with stress exposure to estimate the prophylactic role of iNOS inhibitor.

### Increase of iNOS mRNA expression induced by UCMS was abrogated by the iNOS inhibitor 1400 W

**Figure 2:**
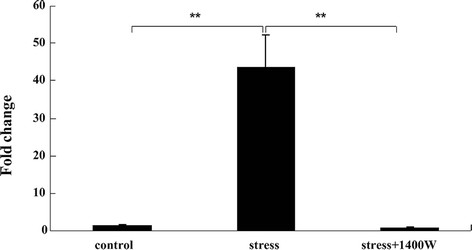
**The specific inducible nitric oxide synthase (iNOS) inhibitor*N*-(3-(aminomethyl)benzyl)acetamidine (1400 W) abrogated the increase of iNOS mRNA expression induced by unpredictable chronic mild stress (UCMS) in the cortex.** UCMS-exposed mice were subjected to various stressors for 4 weeks. Transcript of iNOS was assayed by RT-PCR in the cortex. Values are mean ± SEM (n = 4 in each group). ^**^P <0.01 for each comparison.

**Figure 3:**
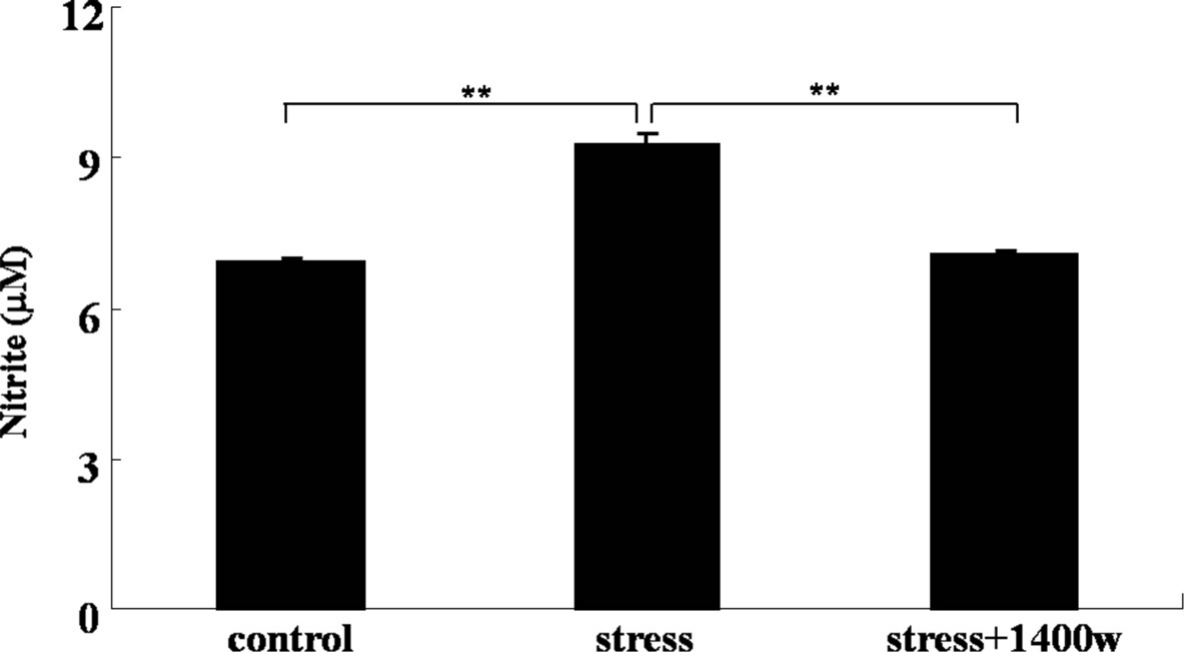
**The specific inducible nitric oxide synthase (iNOS) inhibitor*N*-(3-(aminomethyl)benzyl)acetamidine (1400 W) abrogated the increase of nitrite, a stable NO product.** Mice were subjected to various stressors in the presence or absence of the iNOS inhibitor 1400 W for 4 weeks. Nitrite was assayed by Griess reagent. Data are mean ± SEM (n = 6 for control; n = 6 for stress; n = 5 for stress + 1400 W). ^**^P <0.01 for each comparison.

To examine whether UCMS could induce iNOS expression, we analyzed iNOS mRNA expression after the 4-week UCMS. RT-PCR analysis was performed to investigate whether UCMS induced iNOS mRNA expression in the cortex. As shown in Figure 2, the level of iNOS mRNA expression by 4-week UCMS was significantly enhanced (F_(2, 9)_ = 24.61, *P* < 0.01). Subsequently, the level of the product of iNOS, NO in the plasma was also assayed using Griess reagent. Figure 3 showed that the level of nitrite, the stable end product of NO, in the plasma of UCMS-exposed mice was significantly increased (F_(2, 14)_ = 88.30, *P* < 0.01). Next, the effects of 1400 W on UCMS-induced iNOS expression and NO generation were investigated. 1400 W is the specific iNOS inhibitor exhibiting over 5000-fold or 200-fold greater selectivity for iNOS as compared with eNOS or nNOS. Mice were treated with 1400 W as well as UCMS. The results (Figures 2 and 3) showed that this increase of iNOS mRNA expression and NO production induced by UCMS was abrogated by 1400 W (*P* < 0.01), comparing with that of the stress group.

### Decrease of sucrose preference induced by UCMS was modulated by the iNOS inhibitor 1400 W

**Figure 4:**
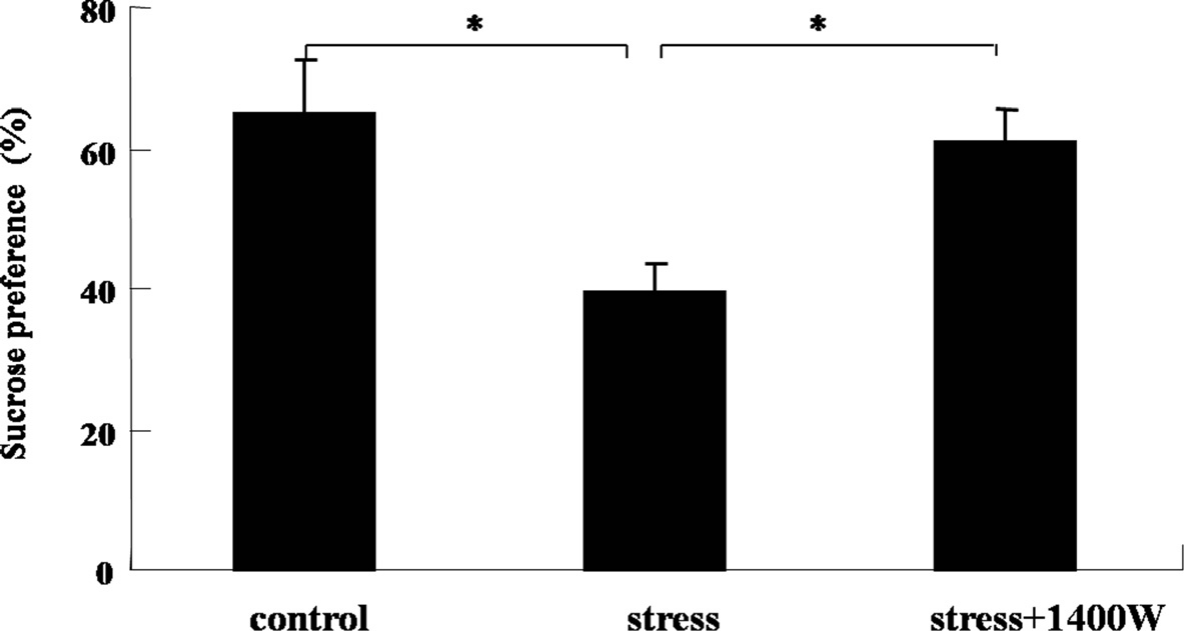
**The specific inducible nitric oxide synthase (iNOS) inhibitor*N*-(3-(aminomethyl)benzyl)acetamidine (1400 W) abrogated the decrease of sucrose preference induced by unpredictable chronic mild stress (UCMS).** Mice were subjected to various stressors in the presence or absence of the iNOS inhibitor 1400 W for 4 weeks. Sucrose preference = sucrose consumption/(sucrose consumption + water consumption) × 100%. Data are mean ± SEM (n = 7 for control; n = 6 for stress; n = 9 for stress + 1400 W). ^*^P <0.05 for each comparison.

To evaluate whether the iNOS pathway is involved in the modulation of the decrease of sucrose preference induced by UCMS, the changes in sucrose preference in UCMS-treated mice with the presence or absence of the iNOS inhibitor 1400 W were measured. The result (Figure 4) showed that the sucrose preference of UCMS-exposed mice decreased remarkably compared to that of the control group (F_(2,19)_ = 5.67, *P* < 0.05). Furthermore, the iNOS inhibitor 1400 W completely abrogated this decrease (*P* < 0.05). There was almost no change in behavior of the mice treated with 1400 W alone (data not shown). Therefore, iNOS might have an effect on UCMS-induced sucrose preference or anhedonia.

### 1400 W abrogated the increase of immobility time in forced swim test by UCMS

**Figure 5:**
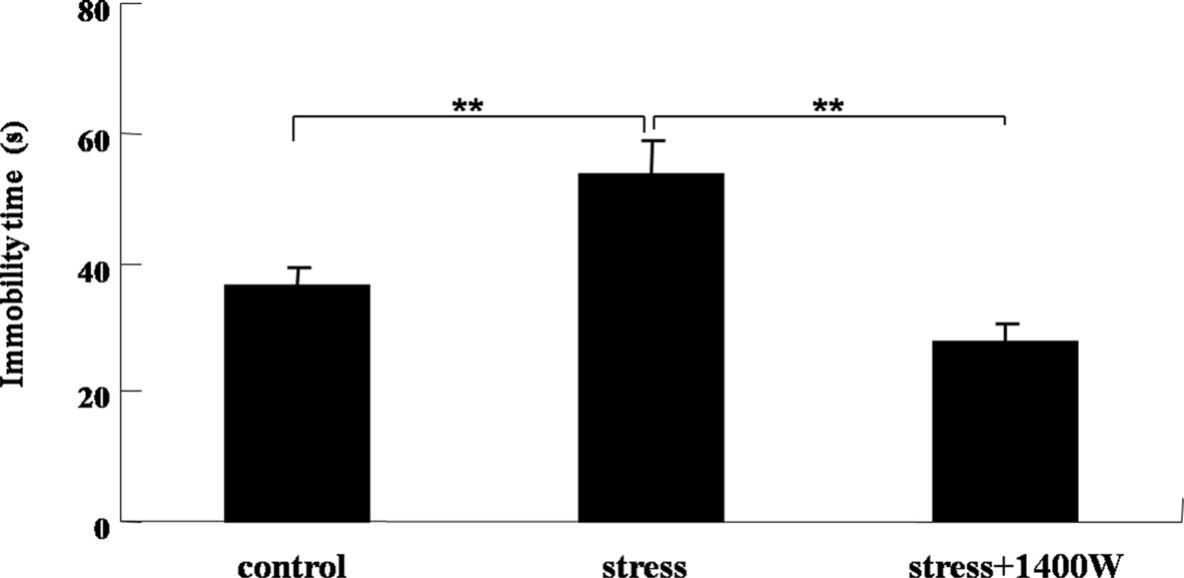
**The specific inducible nitric oxide synthase (iNOS) inhibitor*N*-(3-(aminomethyl)benzyl)acetamidine (1400 W) abrogated the increase of immobility time induced by unpredictable chronic mild stress (UCMS).** Mice were subjected to various stressors in the presence or absence of the iNOS inhibitor 1400 W for 4 weeks. Immobility time was recorded in the forced swim test. Data are mean ± SEM (n = 10 for control; n = 10 for stress; n = 9 for stress + 1400 W). ^**^P <0.01 for each comparison.

The forced swim test (FST) is one of the most commonly used animal behavioral tests for antidepressant screening in the pharmaceutical industry [30-32]. The FST is based on the measurement of the duration that mice and rats stay immobile. Increased immobility in the test is claimed to reflect a helpless or resignation-like state. The immobility time was measured to evaluate whether the iNOS inhibitor 1400 W was involved in FST. After forced swimming for 6 minutes, the immobility time in the last 5 minutes was recorded. As shown in Figure 5, the immobility time of UCMS-exposed mice in the FST increased significantly comparing with that of the control group (F_(2,26)_ = 11.34, *P* < 0.01). Furthermore, the iNOS inhibitor 1400 W completely abrogated this increase (*P* < 0.01). Therefore, iNOS pathway may also play a role in the helpless state of depressive-like behavior.

### 1400 W abrogated the decrease of hole-searching time but had no effect on central time and total travel distance in locomotor activity test by UCMS

**Figure 6:**
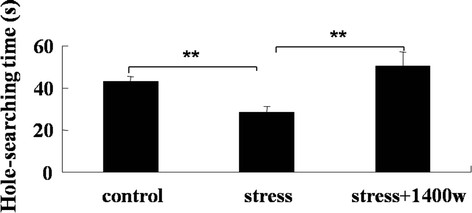
**The specific inducible nitric oxide synthase (iNOS) inhibitor*N*-(3-(aminomethyl)benzyl)acetamidine (1400 W) abrogated the decrease of hole-searching time induced by unpredictable chronic mild stress (UCMS).** Mice were subjected to various stressors in the presence or absence of the iNOS inhibitor 1400 W for 4 weeks. Hole-searching time was recorded in the locomotor activity test. Data are mean ± SEM (n = 8 for control; n = 6 for stress; n = 8 for stress + 1400 W). ^**^P <0.01 for each comparison.

**Figure 7:**
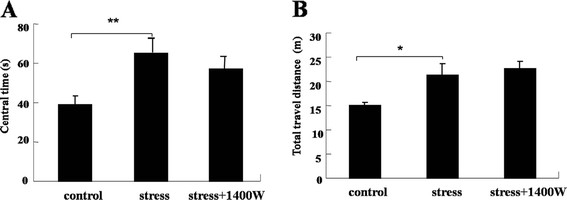
**The specific inducible nitric oxide synthase (iNOS) inhibitor*N*-(3-(aminomethyl)benzyl)acetamidine (1400 W) had no effect on the unpredictable chronic mild stress (UCMS)-induced increase of central time and total travel distance.** Mice were subjected to various stressors in the presence or absence of the iNOS inhibitor 1400 W for 4 weeks. Central time and total travel distance were recorded for 5 minutes: **(A)** central time; **(B)** total travel distance. Data are mean ± SEM (n = 10 for control; n = 10 for stress; n = 9 for stress + 1400 W). ^*^P <0.05, ^**^P <0.01 compared to control.

Hole-searching and central time and total travel distance, which reflect the exploratory behavior, were recorded for 5 minutes in the locomotor activity test to evaluate whether iNOS was involved in the modulation of exploratory behavior. As shown in Figure 6, 4 weeks of UCMS significantly reduced the hole-searching time (F_(2,19)_ = 5.66, *P* < 0.01) compared with that of the control group. Furthermore, the iNOS inhibitor 1400 W completely abrogated this decrease (*P* < 0.01). However, the iNOS inhibitor 1400 W had no effect on the increase of central time and total travel distance induced by UCMS (Figure 7), even though UCMS significantly increased the central time (F_(2,26)_ = 6.37, *P* < 0.01) and total travel distance (F_(2,26)_ = 5.82, *P* < 0.05). Therefore, the iNOS pathway may have certain effects on the exploratory behavior during depressive-like behavior.

### Locomotor activity was not modulated by UCMS and the iNOS inhibitor 1400 W

**Figure 8:**
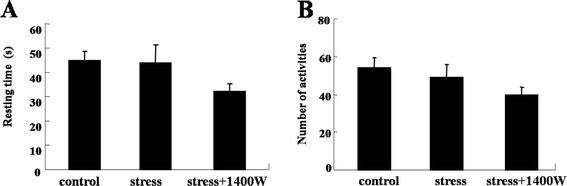
**Unpredictable chronic mild stress (UCMS) and inducible nitric oxide synthase (iNOS) inhibitor*N*-(3-(aminomethyl)benzyl)acetamidine (1400 W) had no effect on resting time and number of activities.** Mice were subjected to various stressors in the presence or absence of the iNOS inhibitor 1400 W for 4 weeks. **(A)** Resting time; **(B)** number of activities. No significant difference was noted for each comparison. Data are mean ± SEM (n = 10 for control; n = 10 for stress; n = 9 for stress + 1400 W).

Locomotor activity is a valid readout of sickness behavior. A sick animal usually exhibits a reduction in locomotor activity. To confirm that UCMS-induced depressive-like behavior was different from the sickness behavior, mice were tested for locomotor deficits. The results showed that there were no significant differences in locomotor activity between the UCMS-exposed mice and those in the control group. These locomotor activities included resting time (Figure 8A, F_(2,26)_ = 1.68) and number of activities (Figure 8B, F_(2,26)_ = 1.70). Furthermore, the iNOS inhibitor 1400 W had no effect on these locomotor activities compared with that of the control group and stress group. Generally speaking, in the absence of any overt signs of sickness behavior, UCMS induced an obvious time-dependent increase in depressive-like behaviors, and the iNOS inhibitor 1400 W could abrogate some of the effects induced by UCMS.

### 1400 W protected cortical neurons from damage induced by UCMS

**Figure 9:**
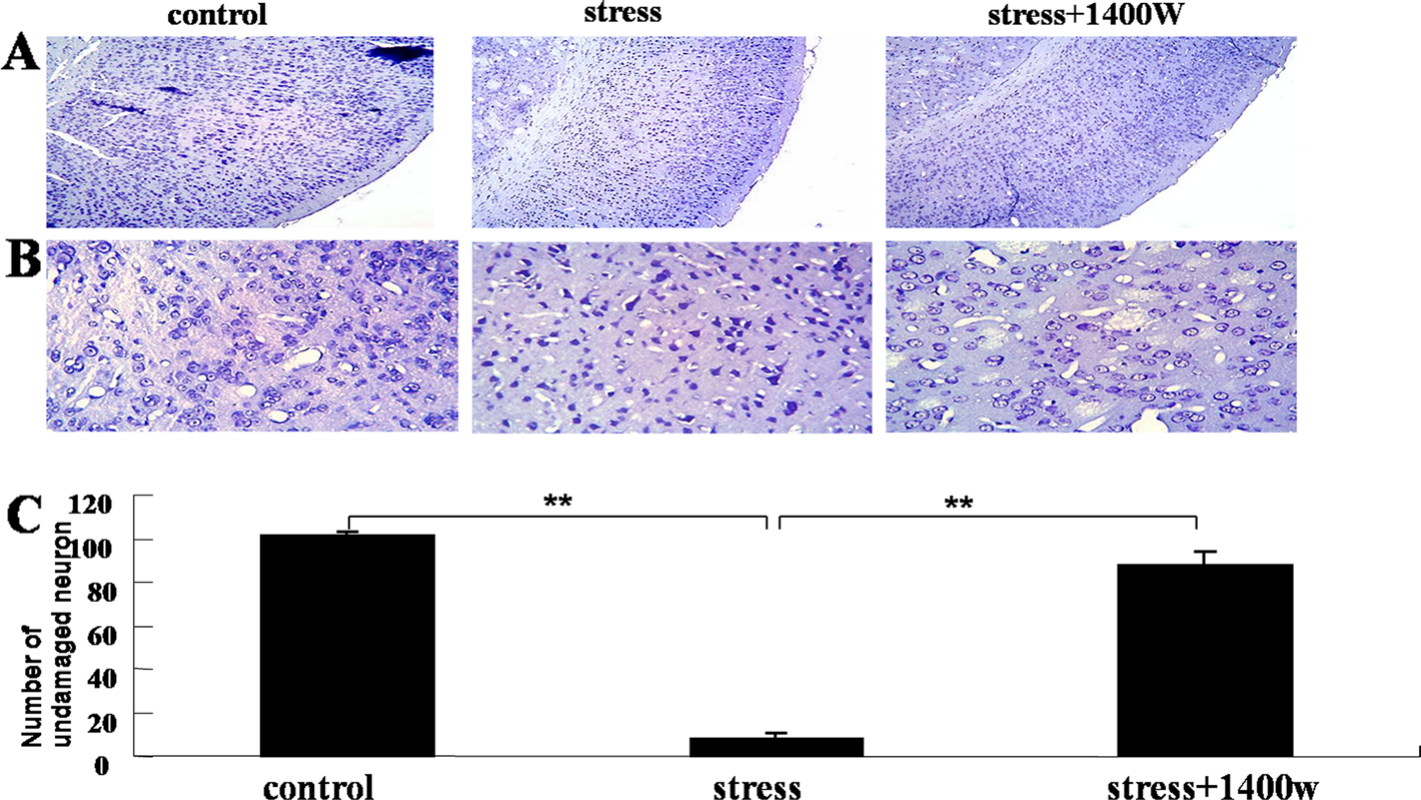
**The specific inducible nitric oxide synthase (iNOS) inhibitor*N*-(3-(aminomethyl)benzyl)acetamidine (1400 W) protected neurons from unpredictable chronic mild stress (UCMS)-induced damage.** Mice were subjected to various stressors in the presence or absence of the iNOS inhibitor 1400 W for 4 weeks. Undamaged neurons in the cerebral cortex were measured by Nissl staining. **(A,B)** Representative slides of Nissl staining at two different magnifications ((A) × 10, (B) × 40). In the control group and stress plus 1400 W group, neurons with round cell bodies, visible nucleus and abundant Nissl bodies were observed, while in stress group, Nissl-positive cells with dark staining in which the nucleus were not discernable were obviously increased. **(C)** Cell counts. The number of undamaged cells in the stress group was significantly lower than that of the control group and stress + 1400 W group. Data are mean ± SEM (n = 3 in each group). ^**^P <0.01 for each comparison.

The effects of chronic stress and iNOS inhibitor pretreatment on cortical neurons were explored. Immunohistochemical analysis for undamaged neurons by Nissl staining was performed. As shown in Figure 9A,B, in the control group and stress plus 1400 W group, neurons with round cell bodies, visible nucleus and abundant Nissl bodies were observed. However in the stress group, Nissl-positive cells with dark staining in which the nucleus were not discernable were obviously increased. Cell counting showed that the number of undamaged cells in the stress group was significantly lower than that of the control group (F_(2,6)_ = 160.11, *P* < 0.01). Furthermore, the iNOS inhibitor 1400 W abrogated this decrease of undamaged neurons (*P* < 0.01). Namely, 1400 W pretreatment protected the cortex neurons from damage induced by UCMS.

## Discussion

The results of the present study demonstrated that chronic stress significantly induced depressive-like behaviors in mice. The levels of iNOS mRNA expression in the cortex and nitrites in the plasma of UCMS-exposed mice were significantly increased compared with that of the control group. The neurons of the cerebral cortex in UCMS-exposed mice were shrunken, together with loss of Nissl bodies. However, the stress-related depressive-like behaviors, the increase of iNOS mRNA expression and nitrites, and neuron damage were remarkably abrogated by pretreating the mice with an iNOS inhibitor (1400 W).

The relationship between NO and depression has been noticed clinically. Plasma nitrate concentrations, an index of NO production, are prominently higher in depressed patients [33]. Additionally, some antidepressants inhibit the enzymatic activity of NOS in animals and humans [34,35]. The above facts implied a possible involvement of NO in depression. In the past few years, several groups have consistently reported that nNOS indeed plays a crucial role in depression. The evidence for nNOS involvement in depression includes nNOS expression increasing in the hippocampus [36] and nNOS inhibition preventing and reversing depressive-like behaviors [37,38]. Recently, Wang *et al*. reported that iNOS also made contributions to the mechanism of depression by using intrahippocampal injections of the iNOS inhibitor aminoguanidine [39]. This research did not explore whether systemic injection of iNOS inhibitor has the similar effect on depressive-like behavior, and how the iNOS inhibitor functions, which is our focus in this study. Mice were treated with UCMS for 4 weeks consecutively in the presence or absence of the specific iNOS inhibitor 1400 W. The results showed that treatment of mice with the iNOS inhibitor 1400 W prevented the decrease of sucrose preference (*P* < 0.05), the increase of immobility time in FST (*P* < 0.01), and the decrease of exploring time in the locomotor activity test (*P* < 0.01) induced by UCMS. These actions are specific to depressive-like behavior and have no effect on locomotor activity such as active time and resting time, which reflects sickness behavior.

In accordance with the change of depressive-like behavior, the iNOS inhibitor 1400 W exhibited a significant protection on neurons compared with UCMS group (*P* < 0.01). UCMS caused the loss of Nissl bodies in the cerebral cortex neurons, and 1400 W pretreatment prevented this loss. Namely, blocking NO abrogated neuronal damage caused by UCMS. This protection by 1400 W might be related to neuronal functional impairment and structural damage caused by excessive NO. In fact, stress-related events including depression are characterized by modifications of oxidative/nitrosative pathways in the brain in response to the activation of inflammatory mediators [1]. It has been shown that repeated and unpredictable stress situations increase generation of reactive oxygen species (ROS) in the brain, which in turn results in oxidative damage in the central nervous system [40,41]. Recent findings indicate a key role for NO and an excess of pro-oxidants in various brain areas is responsible for both neuronal functional impairment and structural damage [42]. Nevertheless, the effects of oxidative stress in this present system require further study, such as the measurement of some oxidative/antioxidative parameters.

Over the last two to three decades, inflammatory depression has attracted increasing attention in the field of depression [43,44]. The main theory of inflammatory depression is that the activation of the inflammatory immune system may influence neurochemicals or damage neurons and contribute to depression [45]. Under physiological conditions, these proinflammatory cytokines enhance neurogenesis. However, excessive or prolonged cytokine exposure may damage the brain (including affecting the metabolism of neurotransmitter and neuropeptide, neuroendocrine and neural plasticity, decreasing neurogenesis, increasing glutamatergic activation, oxidative stress, and induction of apoptosis) [46-50]. Chronic inflammation is often associated with clinical depression [51-53]. Chronic stress dysregulates immunity by overactivating the immune system, leading to low-grade inflammation. iNOS is inflammation inducible. Therefore, we conclude that iNOS may play an important role in the depressive behavior induced by chronic stress exposure. Based on our experimental results, chronic stress leads to a prominent increase of inflammatory mediators (data not shown), which is consistent with reports from other groups [42,54,55]. These results support the notion that stress-related NO (derived from iNOS) may contribute to depressive-like behavior in a mouse model, potentially concurrent with neurodegenerative effects within the cerebral cortex. A dose course is recommended in future studies to further establish the rationale between UCMS-related depressive behavior and iNOS/NO. Additionally, complete measurement of the alteration of iNOS in individual brain regions is also suggested, so that the key regions related to UCMS-induced depression based on iNOS/NO can be identified and located.

## Conclusions

In summary, the present study supports the notion that stress-related NO (derived from iNOS) may contribute to depressive-like behavior in a mouse model, potentially concurrent with neurodegenerative effects within the cerebral cortex. These studies provide new insights into the mechanisms underlying the responses of depression to UCMS. A better understanding of the role of key signaling mediators in depression could aid the development of novel pharmacological agents. Further studies on detailed and in-depth molecular mechanisms of iNOS in UCMS-induced depressive-like behavior are recommended.

## Misc

These two authors contributed equally to this work**.**

## Competing interests

The authors declare that they have no competing interests.

## Authors’ contributions

Y-LP and Y-NL contributed equally to this work. Y-LP established the UCMS depression model, analyzed the results and drafted the manuscript. Y-NL performed the behavioral test, with the help of XW. Y-LP was responsible for analyzing the in vivo component of these experiments. LL performed the immunohistochemical experiments. Y-XW and C-LJ secured funding for the project and helped with the final version of the manuscript. All authors read and approved the final manuscript.
